# Spirit Rappings in the French Academy of Sciences

**Published:** 1854-10

**Authors:** 


### SPIRIT RAPPINGS IN THE FRENCH ACADEMY OF SCIENCES.

Some of the readers of this Journal will remember, that an exposure of the pretensions of certain girls to communication with the spiritual world was made by Profs. Flint, Lee, and Coventry, in Buffalo, some years ago, and that these gentlemen, after a careful investigation of the sounds produced by the "Rappers," published an explanation of the phenomena, which was far from being generally received as satisfactory, at the time. For ourselves, we never for a moment hesitated to receive it as the true explanation of the mysterious sounds. In another part of this number will be found a case, reported to the French Academy, by M. Schiff, in which a female was able to create the rappings by the snapping of the tendon of the *peroneus longus* over the external malleolus. The subject was deemed by the Academy worthy of investigation, and a committee was appointed whose report was published in the *Gazette Hebdomadair* of July 7th. The identity of motion detected by M. Schiff in the young French woman, with that in the Fox girls, at Buffalo, was so striking that Prof. Flint, who was at the time in Paris, felt justified in calling the attention of the Academy to his observations, and he accordingly, through M. Rayer, made the following communication to that learned body. No one can now any longer doubt that the explanation offered by Prof. Flint and his colleagues of spirit-rapping is the true one. We subjoin his letter to M. Rayer.

Paris, June 28, 1854.

To M. A. Rayer, *Member of the Academy of Sciences, etc.*

Sir:—Having seen in the columns of the *Gazette des Hopitaux* notice of an observation and experiment relative to *spiritual rappers*, by Dr. Schiff, recently communicated by you to the Academy of Sciences, I beg leave to ask of you the favor to transmit to the Academy an account of certain observations and experiments relative to this subject, with which I have been connected.

An exposure of this most remarkable imposition—an imposition which had for years baffled efforts at detection—was published in the United States, in the spring of 1851, by myself, in conjunction with two of my colleagues, viz., Dr. Charles B. Coventry and Dr. Charles A. Lee. The basis of the exposure was, that the sounds which it was pretended by the authors of the imposition (several females of a family named Fox) were made by the spirits of the dead, were due to the *voluntary muscles acting on the moveable parts of the body.* This principle embraced both movements of the tendons and articulations; and in the course of my investigations, an instance came under my observation precisely similar to that of the young girl examined by Dr. Schiff, and represented by Dr. S. himself before the members of the Academy.

The exposure referred to was published first in a public newspaper at Buffalo, at the time when the Fox girls were in that city holding levees for persons desirous of having intercourse with their deceased relatives. It was shortly afterward communicated to the medical profession in the pages of the Buffalo Medical Journal, of which I am one of the editors; and, subsequently, issued in a brochure. Neither of these printed papers are at hand, at this moment in Paris, but I solicit the favor of transmitting them to the Academy on my return to America. In the mean time, permit me to state the more important of the facts which they contain, which I am able to do perfectly from memory.

The city where I have resided for several years, Buffalo, State of New York, is in the vicinity of the place whence the imposition emanated. The latter is the city of Rochester, and hence the original spiritual rappers are commonly known, in America, as the Rochester rappers.

After visiting several parts of the country, the Fox girls at length honored Buffalo with a visit, and, influenced by curiosity, I was present at one of their exhibitions. A close examination of the countenances of the operators, and the method of conducting the exhibition, satisfied me that the sounds were dependent on the volition of the younger of the two sisters. In discussing the subject afterward, it seemed to me practicable to arrive at the basis of the exposure stated above by the process of reasoning so often useful in diagnosis, viz., reasoning by exclusion. Assuming that the sounds proceeded from volition, it is evident they must be produced either by the voluntary muscles acting on the moveable parts of the body, or by mechanical contrivances superadded to the body. That the latter were not employed might be inferred from the fact of the sound being heard under almost every variety of circumstance, and often repeated examinations of the clothing, etc., leading to no discovery. Hence, by a logical necessity, the search for the explanation was directed toward the muscles, tendons and articulations, these being the only moveable organs under the control of the will.

By a coincidence very remarkable, and which I should attribute to a special interposition of Providence, had the exposure of the imposition sufficed to arrest its progress, a person who was present at the exhibition at the same time as myself, informed me that his wife was able to produce sounds precisely similar to those by means of which the Fox girls were deceiving the public. On visiting this female, I found his statement to be true; and the identity of the sounds as respects character, and intensity, was verified by my colleagues already named, and by several others. This lady permitted us to examine the mode by which she produced the sounds. She was able to effect at will, readily, and without pain, a lateral displacement of the knee-joint. The movement of the bones upon each other gave rise to a loud noise, which, on one occasion, was distinctly heard through a suite of apartments a distance of a hundred feet. She could produce a sound both in moving the bones from their natural position, and in effecting their return; or by causing the displacement or return to take place slowly, she was able to produce a single instead of a double rap. The movements in either direction could be effected silently and she could graduate the intensity of the sound according to the quickness and force of the movements.

In prosecuting further inquiries, I found another female who possessed an analogous faculty of producing sounds with the knee-joint; several instances of ability to produce them by different joints also came under observation, and, as already stated, I met with an instance in which the raps were made by displacement "*in tendon du muscle long pêronier de la gaîne dans laquelle il glisse en passant derrière la malliole externe.*" An account of the instance last mentioned, as well as of the other instances, is given in the publications made by me on this subject in 1851.

These cases were in themselves sufficient to render almost positive the correctness of the principle upon which the exposure by my colleagues and myself was based, viz., that the sounds were due to the voluntary muscles acting on some of the moveable parts of the body. But the infatuated assurance of the Rochester girls furnished an opportunity of rendering the demonstration of the correctness of this principle as complete as possible. Fearful of losing the notoriety and pecuniary gains which they derived from the imposition, they were induced to challenge us publicly, through the papers, to test the truth of our explanation. This challenge was accepted, and the following is a brief account of the interview which took place, by appointment, in the presence of several distinguished gentlemen. Prior to announcing what kind of experiment we should adopt, the spiritual raps were very frequent; and, at our request, the *spirits* were asked if they would consent to hold communication with us, to which an affirmative answer was interpreted by the girls. Acting then, on a hint received from the lady who was able to produce identical sounds, she assuring us that she could not effect the necessary lateral movements unless her feet rested on the floor or against some firm pillar of support, we requested the *rappers* to take a position in the centre of the room on chairs, with the feet elevated and resting on the heels upon soft ottomans. A row of lights was placed on the floor before them in order to detect any sly mode that might be resorted to of violating the conditions of the experiment, and my colleagues and myself took a position for observation in front. Thus arranged the spirits were asked to fulfil their promise. At our instance they were importuned to adhere to their vow, but no sounds were produced. We waited patiently for nearly an hour, and offered to wait as much longer as was desired, and, indeed, did not consent to relinquish the experiment till the girls themselves declared that they had no expectations of the spirits rapping while they were in that position! Immediately on leaving the chairs and ottomans, and resuming their seats on the sofa, with their feet on the floor, the sounds began to be loud and frequent. On expressing freely our views of the success of the trial, the younger sister began to weep hysterically, and the *seance* was thus terminated.

I wish to add that since my attention was directed to this subject I have met with several persons who were able to produce loud sounds very analogous to the Rochester raps by suddenly and loudly retracting and extending upon the sole of the boot, or shoe, the toes; and it is very probable that the Fox girls, or some of their imitators, may adopt this method, which many, if not all persons may acquire by practice, it not requiring, as do the movements of knee and other joints, a peculiarity of conformation. This method, however involves the same principle, viz., the action of the will on muscles and moveable parts. Among the numerous rappers that have sprang up in different portions of the United States, a great variety of means for producing sounds exterior to the body are resorted to, which, however, are generally easily detected. But sounds produced within the ornanism, by the joints and tendons, were well calculated to elude the discovery of their source, inasmuch as this subject appears hitherto not to have received any scientific examination.

The extent to which spiritual rapping belief has prevailed in the United States offers a sad illustration of the credulity of mankind. Among the ranks of believers are not only the ignorant and persons deficient in mental endowments, but not a few of the educated and persons of high intelligence. A late distinguished incumbent of the bench, and also an ex-governor of one of the territories of the United States are its avowed and ardent advocates. Many persons have adopted opinions and regulated their conduct in most important matters by information which they supposed in this way they received from spirits; and eminent clergymen have sought through this channel for enlightenment as respects the theological tenets which they should receive and inculcate. The results of belief in the imposition have already been very grave. A large number of cases of insanity and suicides occurring in different portions of the country have been fairly attributable to it. It is, therefore, certainly a matter of sufficient importance to claim the attention of scientific bodies. This view of the subject, as well as considerations of a personal nature, have led me to desire, to avail myself of the occasion which has now offered itself to present the facts contained in this brief memoir to the Academy of Sciences.

With great respect, yours,

AUSTIN FLINT, M. D.

